# Effects on Bioaccumulation, Growth Performance, Hematological Parameters, Plasma Components, and Antioxidant Responses in Starry Flounder (*Platichthys stellatus*) Exposed to Dietary Cadmium and Ascorbic Acid

**DOI:** 10.3390/antiox12010128

**Published:** 2023-01-05

**Authors:** Tae-Jun Won, Young-Bin Yu, Jung-Hoon Kang, Jun-Hwan Kim, Ju-Chan Kang

**Affiliations:** 1 Department of Aquatic Life Medicine, Pukyong National University, Busan 48513, Republic of Korea; 2 Institute of Marine Biological Resources, Busan 48059, Republic of Korea; 3 Department of Aquatic Life and Medical Science, Sun Moon University, Asan-si 31460, Republic of Korea

**Keywords:** starry flounder, cadmium, ascorbic acid, metal toxicity, mitigative effect

## Abstract

This study evaluates the toxic effects of dietary Cd and mitigative effects of AsA supplementation by measuring the growth performance, bioaccumulation, hematological parameters, plasma components, and antioxidant responses of Starry flounder (*Platichthys stellatus*). *Platichthys stellatus* (mean weight, 69.5 ± 1.4 g; mean length, 18.2 ± 0.21 cm) was fed with dietary cadmium-ascorbic acid (Cd-AsA) composed of C_0_A_0_, C_0_A_500_, C_0_A_1000_, C_40_A_0_, C_40_A_500_, C_40_A_1000_, C_80_A_0_, C_80_A_500_, and C_80_A_1000_ mg of Cd-AsA per kg diet for four weeks. Our results showed that Cd accumulation significantly increased in proportion to the Cd concentration, where the highest levels were observed in the intestine, followed by the kidney, liver, and gills. Dietary AsA significantly mitigated the Cd accumulation in all tissues, and the reduction in Cd accumulation was proportional to the increase in AsA concentration. Dietary Cd has adverse effects on growth performance (body weight gain, specific growth rate, feed conversion ratio, and hepatosomatic index) and can alter the hematological parameters (red blood cell count, hematocrit, and hemoglobin), plasma components (glucose, total protein, glutamic oxaloacetic transaminase, and glutamic pyruvic transaminase), and antioxidant responses (superoxide dismutase, catalase, glutathione S-transferase, and glutathione). Dietary AsA restored the decreased growth performance parameters and the altered hematological parameters, plasma components, and antioxidant responses caused by the dietary Cd exposure. The results of this study showed that dietary Cd is toxic to *P. stellatus*, while dietary AsA is effective in mitigating the toxic effects of Cd.

## 1. Introduction

Cadmium (Cd) is the most toxic metal produced by natural occurrences (e.g., volcanic emissions and weathering of rocks) and anthropogenic activities (e.g., chemical stabilizers, color pigments, electroplating, and byproducts of metal industries) [1]. In the aquatic environment, Cd exists in the form of ions or compounds by binding with inorganic anions (e.g., Cl^−^, SO_4_^2−^, HCO_3_^−^, and F^−^) and organic ligands (e.g., amino acids, citrate, oxalate, salicylate, fulvic acid, and humic acid) through chelation, adsorption/desorption and precipitation/dissolution [2]. Moreover, Cd interacts with organic environmental pollutants (e.g., benzo(a)pyrene, endosulfan, and deltamethrin) and other metals (e.g., calcium, copper, and zinc), resulting in a synergistic toxic effect [3]. The levels of Cd in aquatic environments have constantly been increasing owing to anthropogenic activities, thereby increasing the potential risk of Cd exposure to aquatic organisms.

Cd exposure can induce the bioaccumulation of Cd in the higher-trophic organism due to its non-biodegradation and long half-life in the body [4]. The absorption of Cd in fish occurs through the skin, gills, and intestines via transcutaneous uptake, respiratory tract, and ingestive intake, respectively. The absorbed Cd can be distributed to various tissues via passive diffusion, carrier-mediated transport, and endocytosis [5]. The pattern of bioaccumulation of Cd in specific tissues depends on various factors, such as exposure factors (exposure pathway, period, and concentration), biological factors (fish species and metabolic activity), metal-related factors (metal type, metal concentration, exposure period, and interaction with other metals), and environmental factors (temperature, pH, salinity, and hardness) [6]. Bioaccumulation monitoring identifies the distribution of certain substances and determines the toxic threshold value on various biological levels, such as tissues and cells [7].

Hematological parameters can be highly affected by Cd toxicity since Cd is transported, distributed, and eliminated through erythrocytes and plasma proteins [8]. In addition, Cd can bind to polyunsaturated fatty acids in the erythrocyte membrane, thereby inducing morphological changes [9]. Moreover, Cd interferes with the process of iron transport by competing with iron, an important element in heme synthesis. This results in iron deficiency, which, consequently, affects hemoglobin production [10]. Cd also interferes with the functions of the hematopoietic system that produces mature erythrocytes by damaging hematopoietic cells and suppressing erythropoietin production [11]. In plasma, Cd can alter the physiological status by binding to various plasma proteins, such as detoxification proteins (metallothionein), redox proteins (selenoproteins), and transport proteins (albumin and transferrin) [8]. 

In addition, Cd competes with many ions, such as calcium, magnesium, and zinc, for their binding sites, thus interfering with ion uptake, thereby disrupting ion homeostasis [12]. Cd exposure affects amino acid metabolism and carbohydrate- and lipid-based metabolic processes. It also affects various enzymes, such as phosphofructokinase, lactate dehydrogenase, and transaminases [13]. The toxicity effects of Cd exposure on hematological parameters lead to physiological changes in fish, which in turn, adversely affects their health status.

Cd exposure indirectly or directly induces the reactive oxygen species (ROS) generation, which in turn, induces the redox imbalance and oxidative damage, inhibits the mitochondrial electron transport chain, and impairs mitochondrial complexes II and III, thereby exacerbating ROS generation, mitochondrial dysfunction, and apoptotic cell death [14]. Additionally, Cd can increase the amount of free iron ions participating in the Fenton reaction, which can increase the number of hydroxyl radicals (•OH) [15] and deplete glutathione (GSH), an abundant intracellular thiol-based antioxidant due to its high affinity for thiols, which can reduce the antioxidant capacity [16]. In fish, ROS generation is controlled by antioxidant defense systems, including enzymatic (e.g., superoxide dismutase [SOD], catalase [CAT], glutathione peroxidase [GPx], glutathione-*S*-transferase [GST]), and non-enzymatic antioxidants (e.g., reduced GSH, ascorbic acid, and α-tocopherol), which neutralize ROS and prevent redox imbalance [17].

_L_-ascorbic acid (AsA) is a vital nutrient in fish diets since they lack _L_-gulonolactone oxidase activity that catalyzes the conversion of L-gulonic acid to AsA. AsA prevents symptoms of AsA deficiency, such as reduced growth rate, impaired feed conversation, skeletal deformities (scoliosis, lordosis, abnormal cartilage of eyes, gills, and fins), and reduced immunity [18]. An adequate supply of AsA not only prevents the symptoms of AsA deficiency but also helps with many biological processes, including physiological homeostasis and redox balance. Moreover, AsA can improve hemoglobin functions by increasing the absorption of iron [19] and is also involved in various synthesis pathways, such as blood vessels, collagen, connective tissues, and bone matrix, which help in wound repair and maintaining bone structure [20]. As an antioxidant, AsA neutralizes ROS such as O_2_^−^ and •OH radicals by donating electrons, thereby maintaining redox homeostasis and protecting oxidative damage [21]. In addition, AsA suppresses the pro-oxidant activity by recycling α-tocopherol, an oxidized form of α-tocopherol. The interaction between AsA and α-tocopherol can effectively prevent lipid peroxidation [22].

The starry flounder, *Platichthys stellatus*, is euryhaline fish that inhabits the marine environments of many countries, including Korea, Japan, and the US states, Alaska and California. In Korea, *P. stellatus* is one of the commercially-farmed fish due to its high salinity tolerance and disease resistance. *P. stellatus* production in Korea has steadily increased from 2377 tones in 2014 to 3373 tones in 2018 [23]. Cd can be present at a relatively high level due to anthropogenic activities, and its accumulation can induce severe physiological disturbances and oxidative stress, whereas AsA supplementation benefits various physiological and antioxidant functions. It is important to mitigate Cd toxicity, in which AsA, an exogenous additive, is likely to play a positive role in detoxification. Previous studies have assessed Cd toxicity and the mitigating effects of AsA on aquatic animals, but there is limited research on *P. stellatus*. Therefore, this study aimed to evaluate the toxic effects of dietary Cd and mitigative effects of AsA supplementation by measuring the growth performance, bioaccumulation, hematological parameters, plasma components, and antioxidant responses of *P. stellatus*.

## 2. Materials and Methods

### 2.1. Experimental Diets

All diets contained 62% white fish meal, 10% casein, 19.42% dextrin, 2% fish oil, 2% squid liver oil, 1% carboxymethylcellulose, 1% vitamin premix (AA-free), 1% mineral premix, and 0.5% choline salt. The Cd premix was prepared with 10 g CdCl_2_ (Sigma Chemical Co., St. Louis, MO, USA) and 340 g α-cellulose and used to design Cd concentrations (0, 40, and 80 mg/kg; C_0_, C_40_, and C_80_) (Table 1). We used _L_-ascorbyl-2-monophosphate (AMP), purchased from WOOSUNGFEED Co., Ltd., Daejeon, Korea, as the AsA source. The AMP premix was made with 50 g AMP and 450 g α-cellulose and used to design AsA concentrations (0, 500, and 1000 mg/kg; A_0_, A_500_, A_1000_). Concomitant with the increase in the amounts of Cd and AMP, the equivalent amount of α-cellulose was reduced to maintain the nutrient ratio. All ingredients were thoroughly mixed using a bakery mixer (Model: KB201; Kimhill Bakery Machinery Co., LTD., Chiayi, Taiwan) and extruded as a 2 mm diameter module using a pellet machine (Bakokyong Commercial Co., Pusan, Republic of Korea). All pellets were left to dry for 48 h at 28 °C, sorted in a plastic bottle, and kept at −20 °C until further use. The actual concentration of Cd in the diet was measured using inductively coupled plasma mass spectrometry (ICP-MS) (ELAN 6600DRC, PerkinElmer, Shelton, CT, USA) and expressed as μg/g. A pure standard Cd solution (PerkinElmer) was used for the standard curve.

### 2.2. Experimental Design

Samples of *P. stellatus* (mean weight, 69.5 ± 1.4 g; mean length, 18.2 ± 0.21 cm; *n* = 540) were purchased from a commercial farm (Gijang, Republic of Korea). Fish were acclimated to four 1000 L cylindrical water tanks (100–130 fish per tank) on the flow-through system for one week and were fed a commercial diet at a ration of 4% body weight per day. After acclimation, 180 fish were chosen at random from four 1000 L tanks and divided into nine groups (*n* = 20) for the experiment according to the Cd-AsA dietary concentrations (C_0_A_0_, C_0_A_500_, C_0_A_1000_, C_40_A_0_, C_40_A_500_, C_40_A_1000_, C_80_A_0_, C_80_A_500_, and C_80_A_1000_) for two periods (two and four weeks), and each group was reared in a separate 500 L cylindrical tank under the experimental conditions (temperature 19.3 ± 0.3 °C, salinity 34.1 ± 0.1%, pH 8.1 ± 0.05, dissolved oxygen 7.36 ± 0.3 mg/L, ammonia 1.05 ± 0.52 μg/L, nitrite 5.2 ± 0.21 μg/L, nitrate 0.59 ± 0.04 mg/L, and phosphate 7.1 ± 0.4 μg/L, photoperiod 12 h light: 12 h dark). Fish were fed the Cd-AsA diet at a ration of 4% body weight per day during the exposure period, and the residuals were removed to avoid the effect of Cd or AsA derived from the dissolution in the rearing water. No mortality was observed throughout the study period. At the end of each exposure period, ten fish per tank were selected and anesthetized in a diluted tricaine methanesulfonate solution (MS-222; Sigma-Aldrich, St. Louis, MO, USA). After anesthesia, fish were weighed to evaluate their growth performance. Blood samples were collected from the caudal vein of the fish using a 1 mL syringe with heparin (Sigma-Aldrich, St. Louis, MO, USA) and centrifuged at 3000× *g* for 10 min at 4 °C to obtain the plasma (Model: Mikro 22R; Hettich GmbH & Co. KG, Tuttlingen, Germany) which was kept at −20 °C until analysis of the plasma components. The intestine, kidney, liver, and gills were dissected and frozen at −80 °C until further use for the analysis of tissue accumulation and antioxidant response. This experiment was repeated three times. All experimental procedures were approved by the Institutional Animal Care and Use Committee of Pukyong National University.

### 2.3. Bioaccumulation Analysis

Tissue samples from the intestine, kidney, liver, and gills were rinsed with ultrapure water and freeze-dried for 48 h to obtain the dry weights of the samples. After freeze-drying, 0.1 g of the tissue samples were digested in 6 mL of nitric acid (70% HNO_3_, Guaranteed Reagent grade) and 2 mL of perchloric acid (70% HClO_4_, Guaranteed Reagent grade) at 28 °C for 4 h. The reagents were then evaporated on a hotplate at 105 °C overnight until a clear solution was obtained. At the end of the digestion, the solutions of the digested samples were filtered through a membrane filter (pore size: 0.2 μm, ADVANTEC, Toyo Roshi Kaisha, Ltd., Tokyo, Japan) to remove impurities. The filtrate was analyzed using ICP-MS (ELAN 6600 DRC, PerkinElmer) equipped with argon gas. Cd bioaccumulation in tissues was calculated using a pure Cd standard solution (PerkinElmer) and expressed as μg/g dry weight.

### 2.4. Growth Performance

The whole body weight of the fish and their livers were measured at the end of each exposure period, and growth performances were calculated using the following equations:

Body weight gain (BWG) (%) = 100 × (W_f_ − W_i_)/(W_i_)

Specific growth rate (SGR) (%) = 100 × (Ln W_f_ − Ln W_i_)/D

Feed conversion ratio (FCR) = F_i_/(W_f_ −W_i_)

Hepatosomatic index (HSI) (%) = 100 × W_L_/W_f_

where W_f_ is the final body weight; W_i_ is the initial body weight; W_L_ is the liver weight; F_i_ is the deed intake; D indicates the days

### 2.5. Hematological Parameters and Plasma Components

Blood samples used to evaluate the total red blood cell (RBC) counts, hemoglobin (Hb), and hematocrit (Ht) were diluted 400-times with Hayem’s diluting solution, and RBC counts were analyzed using a hemocytometer (Improved Neubauer, Paul Marienfeld GmbH & Co. KG, Lauda-Königshofen, Germany). Blood samples were placed into micro-hematocrit capillary tubes (Paul Marienfeld GmbH & Co. KG, Lauda-Königshofen, Germany). The capillary tubes were sealed at one end with a tube sealing compound and subsequently centrifuged at 12,000 rpm for 5 min using a micro-hematocrit centrifuge (Model; 01501, HAWKSLEY & SONS, Ltd., Lancing, UK). Ht values were expressed as percentages. Hb levels were analyzed using a clinical kit (Asan Pharm. Co., Ltd., Seoul, Republic of Korea) according to the manufacturer’s instructions. Plasma components were used to evaluate the level of glucose, total protein, glutamic oxaloacetic transaminase (GOT), and glutamic pyruvic transaminase (GPT), using a clinical kit (Asan Pharm. CO., Ltd.), according to the manufacturer’s instructions. Glucose was analyzed using the GOD/POD method, and the absorbance was measured at 500 nm and expressed as mg/dL. Total protein was quantified using the biuret method, and the absorbance was measured at 540 nm and expressed as g/dL. GOT and GPT were analyzed using the Reitman-Frankel method, and the absorbance was measured at 505 nm and expressed as karmen/mL. 

### 2.6. Antioxidant Responses

The intestine, kidney, liver, and gills were placed in a micro-centrifuge tube (BIOFACT Co., Ltd., Daejeon, Republic of Korea) with cold 0.1 M phosphate buffered solution (PBS, pH 7.4) and stainless bead and were homogenized using a GeneReady Standard homogenizer (Model; BSH-2, BIOFACT CO., Ltd.). The homogenates were centrifuged at 10,000× *g* for 30 min at 4 °C, and the supernatants were used for the analysis of antioxidant responses. The SOD and CAT activity was measured using the SOD Assay Kit (Dojindo Molecular Technologies, Inc., Rockville, MD, USA) and CAT Assay Kit (Cell Biolabs, Inc., San Diego, CA, USA), respectively, according to the manufacturer’s instructions. GST activity and GSH levels in tissues were analyzed as described previously [24]. Quantitative protein content in tissues was performed using Total Protein Assay Kit (Bio-Rad Laboratories, Inc., Seoul, Republic of Korea) according to the manufacturer’s instructions using bovine γ-globulin as a standard. 

### 2.7. Statistical Analysis

Data and statistical analyses were performed using SigmaPlot 12.0 (Systat Software, Inc., Palo Alto, CA, USA) and the SPSS Statistics, version 27 (SPSS Inc., Chicago, IL, USA). The between-groups analysis was performed at two and four weeks using one-way analysis of variance (ANOVA) and Tukey’s multiple-range test and classified as significant differences at *p* values of <0.05. All data were expressed as mean ± standard error (S.E).

## 3. Results

### 3.1. Bioaccumulation

**Figure 1:**
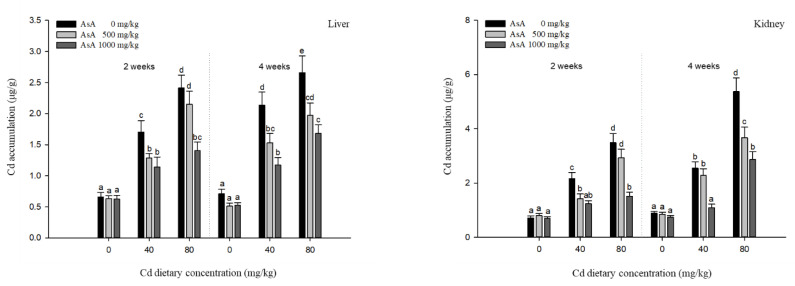

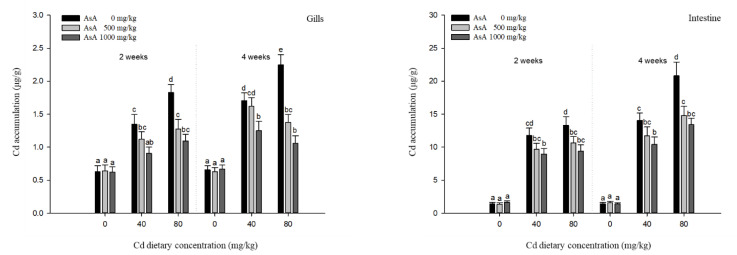
Cadmium accumulation in the liver, kidney, gills, and intestines of starry flounder, *Platichthys stellatus* exposed to dietary Cd-AsA after 2 and 4 weeks. Values are shown as mean ± SE (*n* = 10). The superscript letters indicate the significant differences between groups (*p* < 0.05) at 2 and 4 weeks as determined using Tukey’s multiple range test.

Cd accumulation in the liver, kidney, gills, and intestines of *P. stellatus* exposed to dietary Cd-AsA after two and four weeks is shown in Figure 1. Cd accumulation in the liver, kidney, gills, and intestines in the groups exposed to 40 and 80 mg/kg of dietary Cd was significantly higher than that in the groups exposed to the 0 mg/kg dietary Cd. The accumulation of Cd was significantly proportional to the increase in the Cd concentration. The Cd accumulation profile was the highest in the intestine, followed by the kidney, liver, and gills. Dietary AsA significantly reduced the Cd accumulation at weeks two and four. The Cd accumulation in the liver, kidney, gills, and intestines in the groups exposed to 500 and 1000 mg/kg dietary AsA was significantly lower than that in the group exposed to 0 mg/kg dietary AsA. In particular, compared to 500 mg/kg dietary AsA, the 1000 mg/kg dietary AsA had a significant effect on reducing the Cd accumulation when fish were exposed to 80 mg/kg Cd at two weeks and 40 mg/kg Cd at two and four weeks.

The reduction ratios of the Cd accumulation are presented in Table 2. The mitigative effect of Cd accumulation increased in proportion to the AsA concentration. The 1000 mg/kg dietary AsA showed the maximum reduction in Cd accumulation in all tissues. In the liver, the reduction ratio of Cd accumulation in the groups exposed to Cd_40_A_1000_ and Cd_80_A_1000_ was 33% and 42% at two weeks and 45% and 37% at four weeks, respectively. In the kidney, the reduction ratio of Cd accumulation in the groups exposed to Cd_40_A_1000_ and Cd_80_A_1000_ was 43% and 57% at two weeks and 57% and 47% at four weeks, respectively, which was the maximum level of reduction among the other tissues. In the gills, the reduction ratio of Cd accumulation in the groups exposed to Cd_40_A_1000_ and Cd_80_A_1000_ was 33% and 40% at two weeks and 27% and 53% at four weeks, respectively. In the intestine, the reduction ratio of Cd accumulation in the groups exposed to Cd_40_A_1000_ and Cd_80_A_1000_ was 24% and 29% at two weeks and 26% and 36% at four weeks, respectively.

### 3.2. Growth Performance

**Figure 2:**
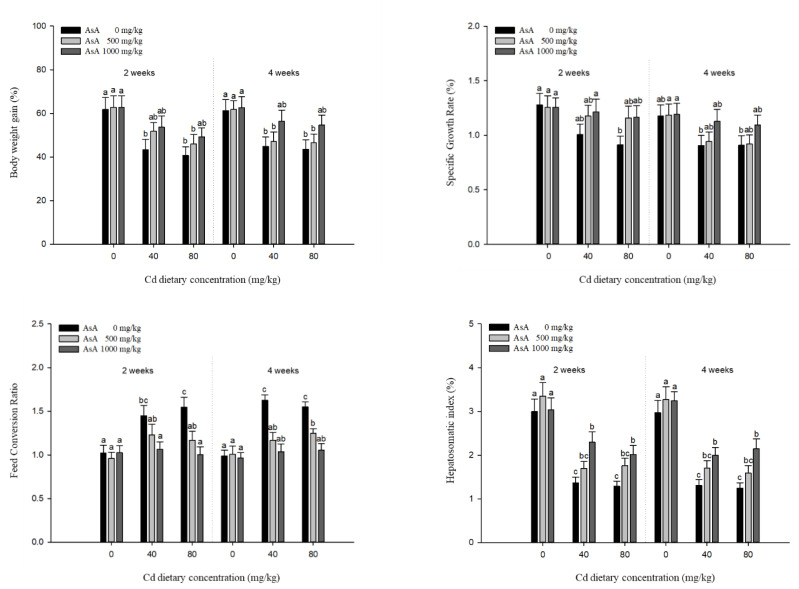
Growth performances of starry flounder, *Platichthys stellatus* exposed to dietary Cd-AsA after 2 and 4 weeks. Values are shown as mean ± SE (*n* = 10). The superscript letters indicate the significant differences between groups (*p* < 0.05) at 2 and 4 weeks as determined using Tukey’s multiple range test.

The growth performance of *P. stellatus* exposed to dietary Cd-AsA for two and four weeks is shown in Figure 2. The growth performance parameters, such as BWG, FCR, SGR, and HSI, were significantly affected compared to the Cd concentration. The BWG, FCR, and HSI of the groups exposed to 40 and 80 mg/kg dietary Cd at two and four weeks were significantly lower than those of the groups exposed to 0 mg/kg dietary Cd, while the FCR showed the opposite effects. However, the dietary AsA mitigated the decrease in growth performance caused by Cd exposure. The BWG and SGR showed no significant difference between the groups exposed to dietary AsA 0, 500, and 1000 mg/kg at two and four weeks but increased to levels close to those of the groups exposed to 0 mg/kg dietary Cd as the AsA concentration increased. The FCR of the groups exposed to 500 and 1000 mg/kg dietary AsA was significantly lower than those exposed to 0 mg/kg dietary AsA at two and four weeks. Moreover, the levels of FCR decreased close to those of the groups exposed to 0 mg/kg Cd with an increase in AsA concentration. The HSI of the groups exposed to 1000 mg/kg dietary AsA was significantly higher than that of the groups exposed to 0 and 500 mg/kg dietary AsA at two and four weeks. These results indicated that dietary AsA mitigated the decrease in the parameters of growth performance due to Cd toxicity.

### 3.3. Hematological Parameters and Plasma Components

**Figure 3:**
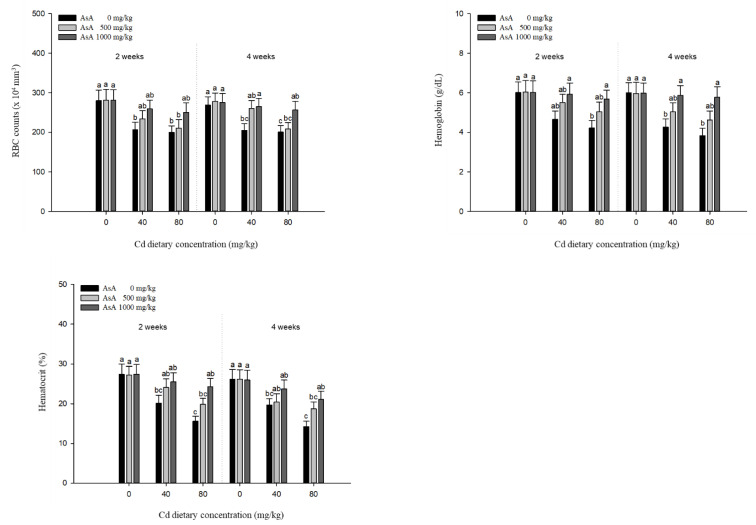
Hematological parameters of starry flounder, *Platichthys stellatus* exposed to dietary Cd-AsA after 2 and 4 weeks. Values are shown as mean ± SE (*n* = 10). The superscript letters indicate the significant differences between groups (*p* < 0.05) at 2 and 4 weeks as determined using Tukey’s multiple range test.

The hematological parameters of *P. stellatus* exposed to dietary Cd-AsA after two and four weeks are shown in Figure 3. Hematological parameters such as RBC counts, Hb, and Ht were significantly decreased in proportion to Cd concentration. RBC counts, Hb levels, and Ht levels of the groups exposed to 40 and 80 mg/kg dietary Cd were significantly lower than those of the groups exposed to 0 mg/kg dietary Cd at two and four weeks, except for the Hb levels of the groups exposed to 40 mg/kg dietary Cd at two weeks. Dietary AsA mitigated the decrease in the levels of the hematological parameters caused by dietary Cd, and its rate was proportional to the AsA concentration. Dietary AsA in a concentration of 1000 mg/kg significantly increased the RBC counts of the groups exposed to 40 and 80 mg/kg dietary Cd at four weeks compared to the group exposed to 0 mg/kg dietary AsA. Except for the group exposed to 40 mg/kg dietary Cd at two weeks, the decreased Hb levels due to 40 and 80 mg/kg dietary Cd were significantly increased by the 1000 mg/kg dietary AsA at two and four weeks compared to the 0 mg/kg AsA. The Ht levels of the groups exposed to 80 mg/kg Cd at two and four weeks were significantly increased by the 1000 mg/kg dietary AsA compared to the 0 mg/kg AsA. 

**Figure 4:**
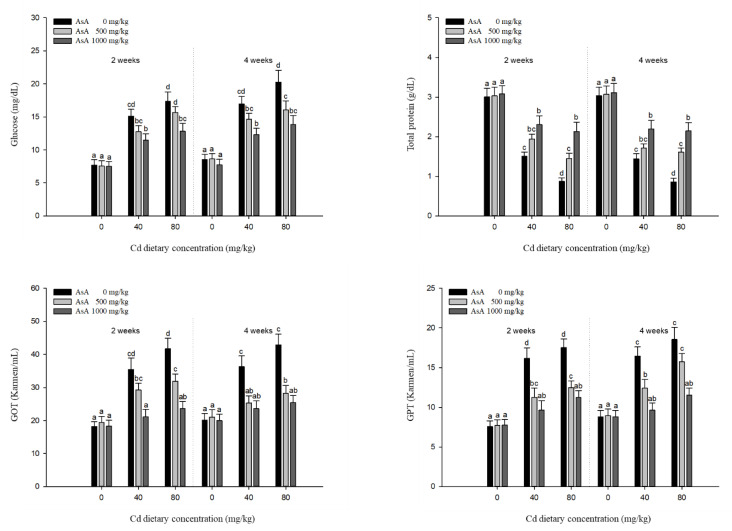
Plasma components of starry flounder, *Platichthys stellatus* exposed to dietary Cd-AsA after 2 and 4 weeks. Values are shown as mean ± SE (*n* = 10). The superscript letters indicate the significant differences between groups (*p* < 0.05) at 2 and 4 weeks as determined using Tukey’s multiple range test.

The plasma components of *P. stellatus* exposed to dietary Cd-AsA after two and four weeks are shown in Figure 4. Glucose, total protein, GOT, and GPT were also significantly affected compared with the Cd concentration. The levels of glucose, GOT, and GPT in the groups exposed to 40 and 80 mg/kg dietary Cd at two and four weeks were significantly higher than those in the groups exposed to 0 mg/kg dietary Cd, but the total protein was significantly lower. Dietary AsA mitigated the altered levels of plasma components caused by dietary Cd, and its rate was proportional to the AsA concentration. The increased levels of glucose, GOT, and GPT due to 40 and 80 mg/kg dietary Cd at two and four weeks were significantly decreased by the 1000 mg/kg dietary AsA compared with 0 mg/kg AsA, whereas the levels of total proteins were significantly increased.

### 3.4. Antioxidant Responses

**Figure 5:**
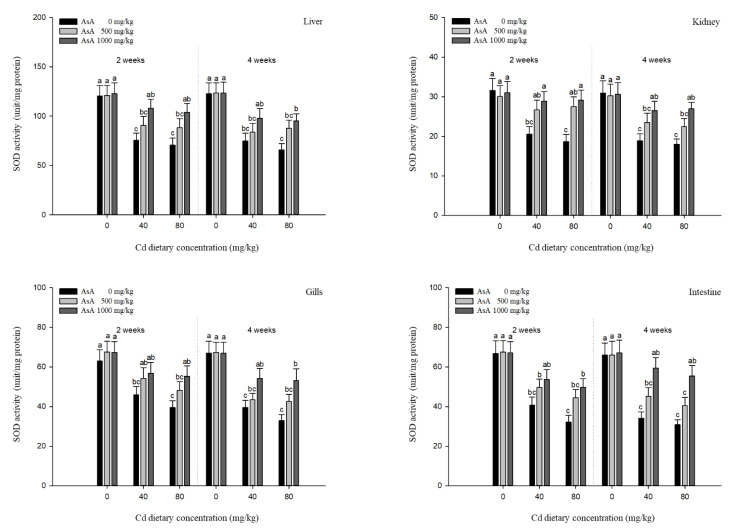
Superoxide dismutase (SOD) activities in the liver, kidney, gills, and intestines of starry flounder, *Platichthys stellatus* exposed to dietary Cd-AsA after 2 and 4 weeks. Values are shown as mean ± SE (*n* = 10). The superscript letters indicate the significant differences between groups (*p* < 0.05) at 2 and 4 weeks as determined using Tukey’s multiple range test.

**Figure 6:**
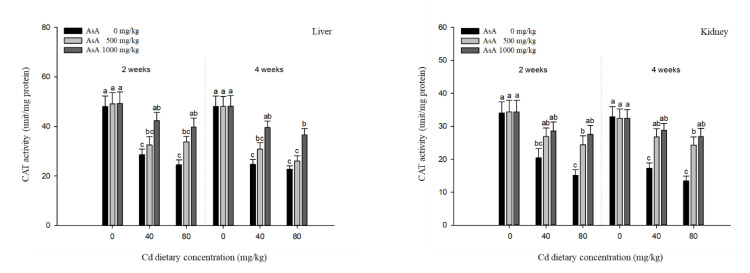

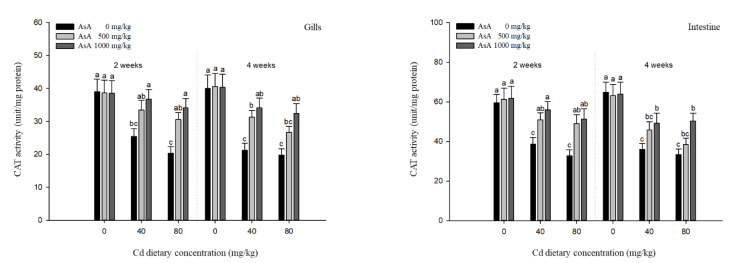
Catalase (CAT) activities in the liver, kidney, gills, and intestines of starry flounder, *Platichthys stellatus* exposed to dietary Cd-AsA after 2 and 4 weeks. Values are shown as mean ± SE (*n* = 10). The superscript letters indicate the significant differences between groups (*p* < 0.05) at 2 and 4 weeks as determined using Tukey’s multiple range test.

**Figure 7:**
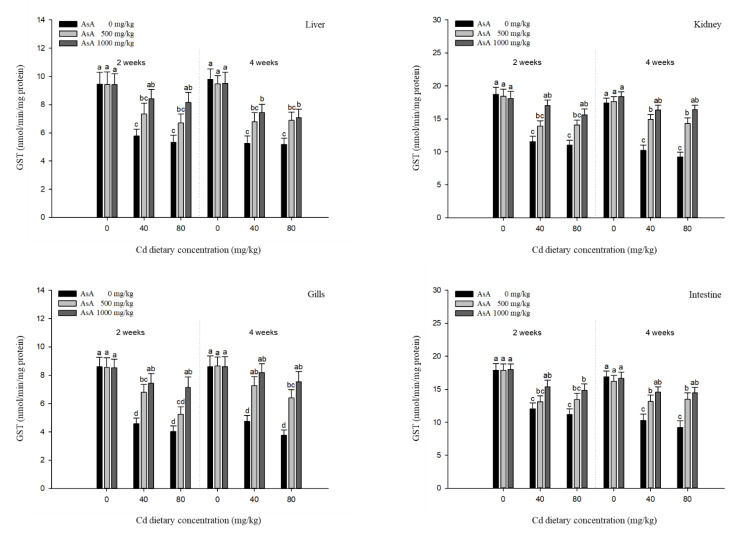
Glutathione-S-transferase (GST) activities in the liver, kidney, gills, and intestines of starry flounder, *Platichthys stellatus* exposed to dietary Cd-AsA after 2 and 4 weeks. Values are shown as mean ± SE (*n* = 10). The superscript letters indicate the significant differences between groups (*p* < 0.05) at 2 and 4 weeks as determined using Tukey’s multiple range test.

**Figure 8:**
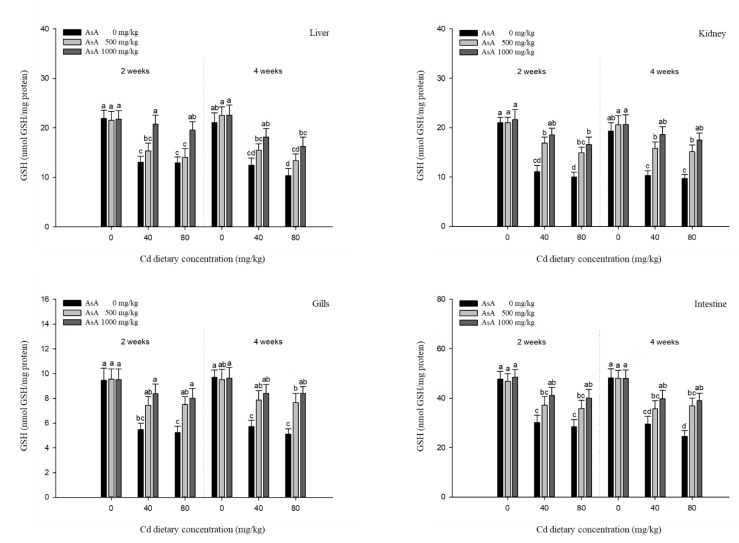
Glutathione (GSH) level in the liver, kidney, gills, and intestines of starry flounder, *Platichthys stellatus* exposed to dietary Cd-AsA after 2 and 4 weeks. Values are shown as mean ± SE (*n* = 10). The superscript letters indicate the significant differences between groups (*p* < 0.05) at 2 and 4 weeks as determined using Tukey’s multiple range test.

The antioxidant responses in the liver, kidney, gills, and intestines of *P. stellatus* exposed to dietary Cd-AsA after two and four weeks are shown in Figure 5, Figure 6, Figure 7 and Figure 8. The SOD and CAT activities in the liver, kidney, gills, and intestines in the groups exposed to 40 and 80 mg/kg dietary Cd at two and four weeks were significantly lower than those in the groups exposed to 0 mg/kg dietary Cd (Figure 5 and Figure 6). Dietary AsA mitigated the decrease in SOD activity caused by dietary Cd, and its rate was proportional to the AsA concentration. The decreased SOD activity in all tissues due to 40 and 80 mg/kg dietary Cd at two and four weeks was significantly increased by the 1000 mg/kg dietary AsA compared with 0 mg/kg AsA. However, there was a decrease in the SOD activity in the gills and intestines at two weeks and liver at four weeks due to the 40 mg/kg dietary Cd. The decreased CAT activity of all tissues due to 40 and 80 mg/kg dietary Cd at two and four weeks was significantly increased by the 1000 mg/kg dietary AsA compared to 0 mg/kg dietary AsA. However, there was an observed decrease in the CAT activity in the kidney due to the 40 mg/kg dietary Cd at two weeks. Similar to the SOD and CAT activities, the GST and GSH levels in the liver, kidney, gills, and intestines in the groups exposed to 40 and 80 mg/kg dietary Cd at two and four weeks were significantly lower than those in the groups exposed to 0 mg/kg dietary Cd (Figure 7 and Figure 8). The dietary AsA mitigated the decreased levels of GST and GSH caused by dietary Cd, and its rate was proportional to the AsA concentration. The decreased GST and GSH levels in all tissues due to the 40 and 80 mg/kg dietary Cd at two and four weeks were significantly increased by the 1000 mg/kg dietary AsA compared with 0 mg/kg dietary AsA.

## 4. Discussion

The analysis of metal accumulation assists in evaluating the toxic effects on fish and determining the routes of metal uptake, biotransformation, and excretion [25]. The gills and intestines are the primary exposure routes for metals, while the accumulation of metals depends on the exposure method, i.e., waterborne or dietary [26]. The dietary Cd is directly accumulated in the intestine, absorbed by the intestinal epithelial cells, and distributed to other organs such as the kidney and liver [5]. The liver and kidney are the organs with the highest Cd accumulation as they detoxify and excrete metals through the induction of metallothionein and metallothionein-like protein [27]. In our result, Cd accumulation was the highest in the intestine, followed by the kidney, liver, and gills. The highest Cd accumulation in the intestine might be due to direct ingestion, whereas the lowest Cd accumulation in the gills might be due to relative accumulation in the kidney and liver, the main target organs of Cd. These findings are in line with other results of previous studies; for example, Kwong et al. [28] reported that the highest level of Cd accumulation in the intestine might be attributed to the intestinal barrier effect on the absorption from the diet. AsA, a representative antioxidant, not only alleviates the toxic effects of Cd but also reduces its accumulation in tissues such as the intestine, kidney, and liver. AsA supplementation reduced the Cd levels in the tissues of common carp, *Cyprinus carpio*, Nile tilapia, and *Oreochromis niloticus* due to its chelating ability [29,30], which can prevent Cd accumulation in the intestine and other organs by producing complexes with Cd. 

These results are consistent with our results, which indicated that Cd accumulation in tissues decreased as the level of AsA increased, suggesting that dietary AsA could assist in reducing the Cd accumulation.

Growth performance is used as an indicator of chronic toxicological investigations as it is sensitive to environmental contaminants [31]. Cd exposure retards the growth performance parameters, such as BWG, SGR, FCR, and HSI in fish. Our results showed that the groups fed with Cd exhibited retarded growth performance proportional to the Cd concentrations, which might be the reduced food intake due to Cd toxicity and increased energy consumption needed for detoxification. Similar to our results, several studies reported that the growth retardation due to Cd toxicity was the restriction of food intake and the elevated metabolic cost for detoxification, which has been observed in other fish species, such as yellow catfish, *Pelteobagrus fulvidraco* [32], and rainbow trout, *Oncorhynchus mykiss* [33]. In contrast, our results showed that AsA supplementation ameliorated the harmful effects of Cd on growth performance proportional to the increasing levels of AsA. AsA prevents the reduction in the metabolic activity and growth rate due to Cd exposure [31] and improves iron absorption by reducing Fe^3+^ to Fe^2+^ and the formation of stable chelates with iron [34]. The effect of AsA on iron absorption may be one of the positive effects on growth performance, compared to the fact that Cd interferes with iron absorption in the intestine. Considering the increase in HSI compared to that in the groups treated with Cd alone, AsA prevented the energy loss due to Cd toxicity. Our results showed that AsA supplementation improved the food intake performance and reduced the toxic effects of Cd on growth performance. 

The analysis of hematological parameters is an important indicator for evaluating the health status of fish, as it serves as a useful and reliable indicator of physiological and pathological changes [35]. It can provide the status of fish regarding the level of oxidative stress, metabolic disorders, reproductive dysfunctions, and disease since blood acts as a medium for intercellular transport and is in contact with various organs and tissues [36]. Cd toxicity causes impairment in erythropoiesis, reduction in the life span of erythrocytes, disequilibrium of osmotic pressure in erythrocytes, and disturbance of plasma electrolyte metabolism [37,38]. A decrease in hematological parameters due to Cd toxicity has been observed in other fish species, such as *O. niloticus* [38] and gibel carp, *Carassius auratus gibelio* [39]. Similar to other reports, our results showed that Cd exposure induced a significant reduction in RBC counts, Hb concentration, and Ht levels compared to the groups exposed to dietary Cd at the 0 mg/kg AsA. However, the groups of *P. stellatus* exposed to dietary AsA showed a significant increase in RBC counts, Hb concentration, and Ht levels, thereby mitigating the toxic effects of Cd on the hematological parameters. AsA prolongs the life span of erythrocytes by protecting the lipid peroxidation of unsaturated fatty acids in the cell membrane from metal toxicity [29]. Moreover, AsA improves hemoglobin function by increasing the absorption of iron and plays an important role in cellular respiration [19]. These effects of AsA ameliorate the altered hematological parameters due to metal toxicity (e.g., chromium and lead) [39,40]. Our results revealed that AsA supplementation resulted in a significant increase in RBC counts, Hb concentration, and Ht levels, which, consequently, helped to mitigate the toxic effects of Cd on different hematological parameters.

The analysis of plasma components was used to evaluate the potential adverse effects of metal exposure on the fish stress response, hepatic function, and hydromineral balance [41,42]. Metal exposure decreases the stored glycogen via catecholamine stimulation, which, consequently, increases blood-glucose levels to supply the energy needed under metal-induced stress [43]. When glycogen reserves are depleted due to energy usage against metal-induced stress, the tissue protein supplies keto-acids via the deamination of amino acids [44]. Alterations in total protein levels can occur during liver damage, dysfunction of amino acid synthesis, and denaturation of proteins [36]. The level of total protein in fish exposed to metal toxicity might be reduced due to energy diversification to compensate for the energy demands against toxic stress [45]. In this study, dietary Cd increased the level of glucose and decreased the level of total protein, which might be due to the compensatory mechanism for the increased energy requirement against Cd-induced stress. In contrast, dietary AsA reduced the level of glucose and increased the level of total protein, suggesting that AsA reduced the toxic stress caused by dietary Cd. Transaminases, such as GOT and GPT, are mainly present in hepatocytes and are involved in amino acid and protein metabolism [39]. GOT and GPT leak into the bloodstream when the liver is damaged, and therefore, these enzymes are used as indicators of liver damage [46]. The elevated activity of transaminases indicates increased protein breakdown to deal with energy demand under metal-induced stress [47]. In our study, dietary Cd significantly elevated GOT and GPT levels, suggesting that Cd exposure leads to hepatic damage. In contrast, dietary AsA decreased the elevated levels of GOT and GPT, indicating that AsA mitigated the Cd-induced stress. 

Analysis of antioxidant responses is an indirect method for assessing oxidative stress in tissues [48]. The antioxidant mechanism in fish exposed to toxic substance appear in two stages, of which at the first stage, antioxidant systems prevent the excessive ROS generation, while at the second stage, the antioxidant system is damaged due to the continuous ROS generation, and consequently, oxidative stress occurs [49]. 

SOD and CAT activities act as the first defense mechanism in the antioxidant system; SOD catalyzes the detoxification of the O_2_^−^ radical to H_2_O_2_, which is subsequently converted into H_2_O and O_2_ by CAT. Cd exposure induces the decrease of antioxidant enzyme activities by inducing ROS production, resulting in oxidative stress. Cd decreased SOD and CAT activities in the liver of *C. carpio*, which might be due to the imbalance between ROS and the antioxidant capacity of Cd [50]. Zebrafish, Danio rerio, exposed to Cd, showed decreased SOD and CAT activity due to the inhibition of antioxidant enzymes [51]. Our results showed that both SOD and CAT activities in *P. stellatus* were significantly reduced in a dose-dependent manner after Cd exposure. These results suggest that excessive ROS production due to Cd toxicity causes an imbalance between antioxidants and ROS, thereby inhibiting SOD and CAT activities. 

GST comprises a group of phase II enzymes that facilitate the excretion of endogenous (e.g., lipid peroxides and oxidative stress products) and exogenous (e.g., toxic metals and pesticides) substrates by binding to the conjugates of GSH with electrophilic metabolites [52]. GSH is composed of γ-glutamate-cysteine-glycine and exists mostly intracellularly in fish [53]. GSH is not only involved in enzyme activity, protection of cells, and synthesis of proteins but also has a strong affinity for most metals, such as mercury, lead, and Cd, which bind to the SH groups of cysteine [54]. Cd exposure influences GST activity and GSH levels in fish tissues. For instance, Dabas et al. [55,56] reported that Cd exposure increased the GST activity in the liver, kidney, and gills of *C. punctatus* and GST activity in the liver and kidney of Labeo rohita, both of which were due to the conjugate reactions that occur in phase II of xenobiotic metabolism. Cellular GSH levels in fish exposed to Cd have been reported to decrease in many studies. The Japanese flounder, *Paralichthys olivaceus*, exposed to Cd showed a decrease in GSH levels in the gills and liver, which might be due to the high Cd accumulation in the cell and the formation of the GS-metal complex [57]. GSH levels in the liver of *O. niloticus* exposed to Cd decreased during the 40 days exposure period, except at day 20, which indicated that changes in GSH metabolism were attributed to oxidative stress and associated with the exposure period [49]. Our results showed that both GST and GSH levels in all tissues of *P. stellatus* were significantly decreased in a dose-dependent manner following Cd exposure. The decreased GST and GSH levels in tissues might be due to the direct conjugation of GSH with Cd and the oxidative stress caused by continuous Cd exposure.

The non-enzymatic antioxidant, AsA, deactivates the free radicals or scavenges the extracellular and intracellular ROS, which, consequently, protects the cells from oxidative stress [58]. Several studies have demonstrated that AsA supplementation could be used as an effective antioxidant against various metal toxicities (e.g., Cd, lead, and copper) that induce oxidative stress. Al-Anazi et al. [29] reported that AsA could alleviate Cd toxicity by reducing SOD and enhancing CAT activity in the liver and kidney of *O. niloticus*, and these results were due to the direct reaction of AsA with superoxide hydroxyl radicals and singlet oxygen. Kim and Kang [41] reported that AsA supplementation attenuated the increased antioxidant responses (e.g., SOD, GST, and GSH levels) in the liver and gills of rockfish, *Sebastes schlegelii*, exposed to dietary lead. Vijayavel et al. [59] also demonstrated that administration of AsA reduced the copper stress by increasing the SOD activity, CAT activity, and GSH levels in thornfish, *Terapon jarbua*, thus inhibiting the continuous ROS production and assisting in maintaining the normal status. Similar to previous studies, our results showed that AsA supplementation mitigated the antioxidant responses (e.g., SOD, CAT, GST, and GSH levels) in a dose-dependent manner and restored the antioxidant responses to control values. These results suggest that AsA supplementation mitigated the Cd-induced toxicity in terms of SOD, CAT, GST, and GSH levels in *P. stellatus*. 

## 5. Conclusions

Our results showed that dietary Cd and AsA had toxic and mitigative effects on the physiological and biochemical functions in a dose-dependent manner. Dietary Cd resulted in the highest accumulation of Cd in the intestine, followed by the liver, kidneys, and gills. Dietary Cd has adverse effects on growth performance (BWG, SGR, FCR, and HSI), causing alterations in hematological parameters (RBC counts, Ht, and Hb) and plasma components (glucose, total protein, GOT, and GPT). In addition, dietary Cd decreased the antioxidant responses (SOD, CAT, GST, and GSH). In contrast, dietary AsA decreased Cd accumulation in proportion to an increase in AsA concentration. The altered growth performance, hematological parameters, plasma components, and antioxidant responses due to Cd toxicity were mitigated by dietary AsA. In conclusion, the results of this study indicate that dietary Cd exerts toxic effects on the growth performance, hematological parameters, plasma components, and antioxidant responses of *P. stellatus*. Moreover, the AsA supplementation enhanced the antioxidant enzyme activities, and the high levels of AsA supplementation were effective in mitigating Cd-induced bioaccumulation and other toxic effects.

## Figures and Tables

**Table 1:** Formulation of the experimental diet (% dry matter).

Ingredients (%)	Cd-AsA Concentration (mg/kg)								
	C_0_A_0_	C_0_A_500_	C_0_A_1000_	C_40_A_0_	C_40_A_500_	C_40_A_1000_	C_80_A_0_	C_80_A_500_	C_80_A_1000_
White fish meal ^1^	62	62	62	62	62	62	62	62	62
Casein ^2^	10	10	10	10	10	10	10	10	10
Dextrin ^3^	19.42	19.42	19.42	19.42	19.42	19.42	19.42	19.42	19.42
Fish oil ^4^	2	2	2	2	2	2	2	2	2
Squid liver oil ^5^	2	2	2	2	2	2	2	2	2
Carboxymethylcellulose ^6^	1	1	1	1	1	1	1	1	1
α-Cellulose ^7^	1.08	0.88	0.68	0.94	0.74	0.54	0.8	0.6	0.4
Vitamin Premix (AMP-free) ^8^	1	1	1	1	1	1	1	1	1
Mineral Premix ^9^	1	1	1	1	1	1	1	1	1
Coline salt ^10^	0.5	0.5	0.5	0.5	0.5	0.5	0.5	0.5	0.5
Cadmium chloride ^11^	0	0	0	0.14	0.14	0.14	0.28	0.28	0.28
_L_-ascorbyl-2-monophosphate ^12^	0	0.2	0.4	0	0.2	0.4	0	0.2	0.4
Total	100	100	100	100	100	100	100	100	100
Actual Cd levels (mg/kg)	0.11	0.12	0.11	38.87	38.52	38.44	64.14	64.35	64.28

^1^ Dajeon Co., Ltd., Pusan, Republic of Korea. ^2^ The Feed Co., Ltd., Pusan, Republic of Korea. ^3^ TS Co., Ltd., Incheon, Republic of Korea. ^4^ Sigma Chemical Co., St. Louis, MO, USA. ^5–7^ Sigma, USA. ^8^ Vitamin Premix (AsA-free) (mg/kg diet): dl-calcium pantothenate, 368; Choline chloride, 10; Inositol, 400; Menadione, 1800; Nicotinamide, 1030; Pyridoxine·HCl, 88; Riboflavin, 380; Thiamine mononitrate, 115; dl-a-tocopherol acetate, 210; Retinyl acetate, 38; Biotin, 10; Folic acid, 20; Cyanocobalamin, 1.3; Cholecalciferol, 13.2. ^9^ Mineral Premix (g/kg): Ferrous Fumarate, 12.5; Dried Ferrous sulfate, 20; Manganese Sulfate, 11.25; Dried Cupric Sulfate. 1.25; Cobaltous sulfate, 0.75; Zinc sulfate, 13.75; Calcium iodate, 0.75; Magnesium Sulfate, 80.2; Aluminum Hydroxide, 0.75. ^10^ Kofavet Co., Ltd., Ulsan, Republic of Korea. ^11^ Sigma Chemical Co., St. Louis, MO, USA. ^12^ Woosung Co., Ltd., Daejeon, Republic of Korea.

**Table 2:** Reduction ratio of Cd accumulation by AsA supplementation.

Tissue	Exposure Period	Dietary Cd-AsA (mg/kg)	Bioaccumulation (μg/g)	Reduction Ratio (%)
Liver	2 weeks	C_40_A_0_	1.70 ± 0.19	-
		C_40_A_500_	1.28 ± 0.07	25
		C_40_A_1000_	1.14 ± 0.16	33
		C_80_A_0_	2.41 ± 0.20	-
		C_80_A_500_	2.15 ± 0.21	11
		C_80_A_1000_	1.40 ± 0.14	42
	4 weeks	C_40_A_0_	2.13 ± 0.21	-
		C_40_A_500_	1.53 ± 0.15	28
		C_40_A_1000_	1.17 ± 0.12	45
		C_80_A_0_	2.66 ± 0.27	-
		C_80_A_500_	1.97 ± 0.20	26
		C_80_A_1000_	1.68 ± 0.14	37
Kidney	2 weeks	C_40_A_0_	2.16 ± 0.22	-
		C_40_A_500_	1.42 ± 0.18	34
		C_40_A_1000_	1.24 ± 0.11	43
		C_80_A_0_	3.48 ± 0.35	-
		C_80_A_500_	2.93 ± 0.31	16
		C_80_A_1000_	1.51 ± 0.14	57
	4 weeks	C_40_A_0_	2.55 ± 0.24	-
		C_40_A_500_	2.27 ± 0.24	11
		C_40_A_1000_	1.09 ± 0.12	57
		C_80_A_0_	5.37 ± 0.50	-
		C_80_A_500_	3.67 ± 0.40	32
		C_80_A_1000_	2.87 ± 0.28	47
Gills	2 weeks	C_40_A_0_	1.35 ± 0.14	-
		C_40_A_500_	1.12 ± 0.11	17
		C_40_A_1000_	0.90 ± 0.10	33
		C_80_A_0_	1.83 ± 0.12	-
		C_80_A_500_	1.27 ± 0.15	31
		C_80_A_1000_	1.09 ± 0.10	40
	4 weeks	C_40_A_0_	1.71 ± 0.12	-
		C_40_A_500_	1.62 ± 0.13	5
		C_40_A_1000_	1.25 ± 0.14	27
		C_80_A_0_	2.25 ± 0.15	-
		C_80_A_500_	1.38 ± 0.12	39
		C_80_A_1000_	1.06 ± 0.11	53
Intestine	2 weeks	C_40_A_0_	11.77 ± 1.11	-
		C_40_A_500_	9.71 ± 0.89	18
		C_40_A_1000_	8.96 ± 0.84	24
		C_80_A_0_	13.29 ± 1.30	-
		C_80_A_500_	10.66 ± 0.98	20
		C_80_A_1000_	9.39 ± 0.94	29
	4 weeks	C_40_A_0_	14.04 ± 1.15	-
		C_40_A_500_	11.71 ± 1.34	17
		C_40_A_1000_	10.45 ± 1.11	26
		C_80_A_0_	20.82 ± 2.08	-
		C_80_A_500_	14.80 ± 1.39	29
		C_80_A_1000_	13.42 ± 0.96	36

## Data Availability

The data presented in this study are available in the article.
